# A Case of Laxative-Induced Severe Hypermagnesemia in a Peritoneal Dialysis Patient

**DOI:** 10.7759/cureus.81923

**Published:** 2025-04-08

**Authors:** Kenta Torigoe, Yuta Ikemi, Emiko Otsuka, Kiyokazu Tsuji, Ayuko Yamashita, Shinichi Abe, Mineaki Kitamura, Takahiro Takazono, Noriho Sakamoto, Kumiko Muta, Hiroshi Mukae, Tomoya Nishino

**Affiliations:** 1 Nephrology, Nagasaki University Hospital, Nagasaki, JPN; 2 Respiratory Medicine, Nagasaki University Graduate School of Biomedical Sciences, Nagasaki, JPN

**Keywords:** end-stage renal disease (esrd), hypermagnesemia, laxative, peritoneal dialysis (pd), serum magnesium

## Abstract

A 55-year-old man undergoing peritoneal dialysis (PD) was transferred from a rehabilitation facility to our hospital due to altered consciousness and respiratory distress. Upon arrival, he presented with a Glasgow Coma Scale score of E3V5M6 and hypercapnia with a pCO₂ of 54.6 mmHg. His reduced urine output raised concerns for uremia secondary to diminished residual renal function, prompting the urgent initiation of hemodialysis. However, subsequent investigations revealed that he had been taking magnesium oxide (1980 mg/day) as a laxative and that his serum magnesium level was markedly elevated at 8.4 mg/dL, consistent with hypermagnesemia. Magnesium toxicity, impaired consciousness, and hypercapnia improved after hemodialysis and the cessation of magnesium oxide. The patient was ultimately switched back to PD without recurrence of hypermagnesemia and was discharged. It is important to note that magnesium clearance in patients undergoing PD relies primarily on residual renal function; thus, in patients with diminished residual renal function, the use of magnesium-containing laxatives may precipitate severe hypermagnesemia. Careful consideration is warranted when prescribing magnesium-containing laxatives in patients with diminished residual renal function, even those undergoing PD.

## Introduction

Peritoneal dialysis (PD), along with hemodialysis and kidney transplantation, is a major renal replacement therapy. It offers advantages such as home-based treatment and better preservation of residual kidney function [1]. However, similar to non-dialysis chronic kidney disease (CKD) and hemodialysis, PD is associated with various complications.

Electrolyte abnormalities are major complications of PD. For example, hypokalemia and hypomagnesemia occur due to gastrointestinal loss, malnutrition, and removal during PD [2]. Furthermore, hypermagnesemia has also been observed in patients undergoing PD [3]. Severe hypermagnesemia can present with a wide range of symptoms, including muscle weakness, hypotension, bradycardia, respiratory distress, and altered consciousness [4].

In general, the causes of hypermagnesemia are diverse, but increased magnesium intake is a major concern. In particular, magnesium-containing laxatives are a risk for hypermagnesemia. Special caution is needed when there is impaired magnesium excretion due to reduced renal function or increased intestinal absorption of magnesium caused by conditions such as inflammatory bowel disease or constipation [4]. However, in patients with PD, magnesium is cleared by both residual renal function and PD; therefore, reports of severe hypermagnesemia in these patients are limited.

In this case report, we present the case of a patient with PD who developed severe hypermagnesemia, altered consciousness, and respiratory distress owing to laxative use.

## Case presentation

**Figure 1:**
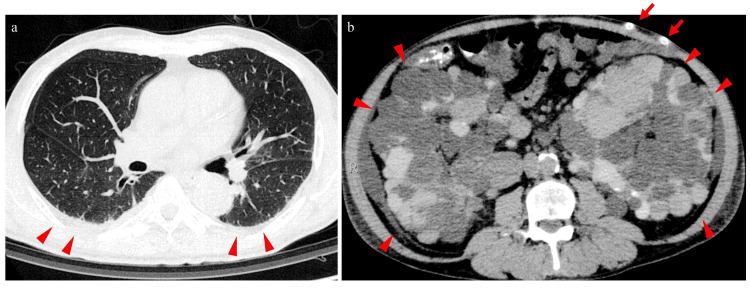
Computed tomography findings at admission (a) Chest computed tomography showed no notable abnormalities except for mild pleural effusion. (b) Abdominal computed tomography showed no notable abnormalities except for the presence of a peritoneal dialysis catheter (arrows) and numerous renal cysts (arrowheads).

The patient was a 55-year-old Japanese man who had been undergoing PD for end-stage renal disease (ESRD) due to polycystic kidney disease for one year and six months. The patient was maintained on automated peritoneal dialysis (APD). One month before being transferred to our hospital, he experienced a left putaminal hemorrhage that was treated conservatively with antihypertensive therapy. He was admitted to a rehabilitation hospital where APD was continued. During hospitalization, his daily urine output decreased from 500 to 200 mL, and his serum creatinine level increased from 15 to 17 mg/dL, raising concerns regarding declining residual kidney function. The day before his transfer to our hospital, he experienced dyspnea and somnolence, prompting a referral for further evaluation. Upon admission to our hospital, his Glasgow Coma Scale (GCS) score was E3V5M6, indicating impaired consciousness. His vital signs were as follows: temperature of 36.9°C, blood pressure of 141/82 mmHg, pulse rate of 98 beats/minute, and SpO₂ of 98% (on 2 L/minute of nasal oxygen). The patient reported difficulty in breathing, and physical examination revealed right hemiparesis as a sequela of the left putaminal hemorrhage. Laboratory tests revealed elevated blood urea nitrogen (45 mg/dL) and creatinine (17.86 mg/dL). Venous blood gas analysis revealed a pH of 7.387, pCO₂ of 54.6 mmHg, and HCO₃⁻ of 32.1 mmol/L, indicating hypercapnia. Non-contrast computed tomography (CT) of the head demonstrated that the hematoma in the left putamen had decreased in size. Chest and abdominal CT tomography revealed multiple cysts in both kidneys and the presence of a PD catheter, with no other significant findings (Figure 1).

**Figure 2:**
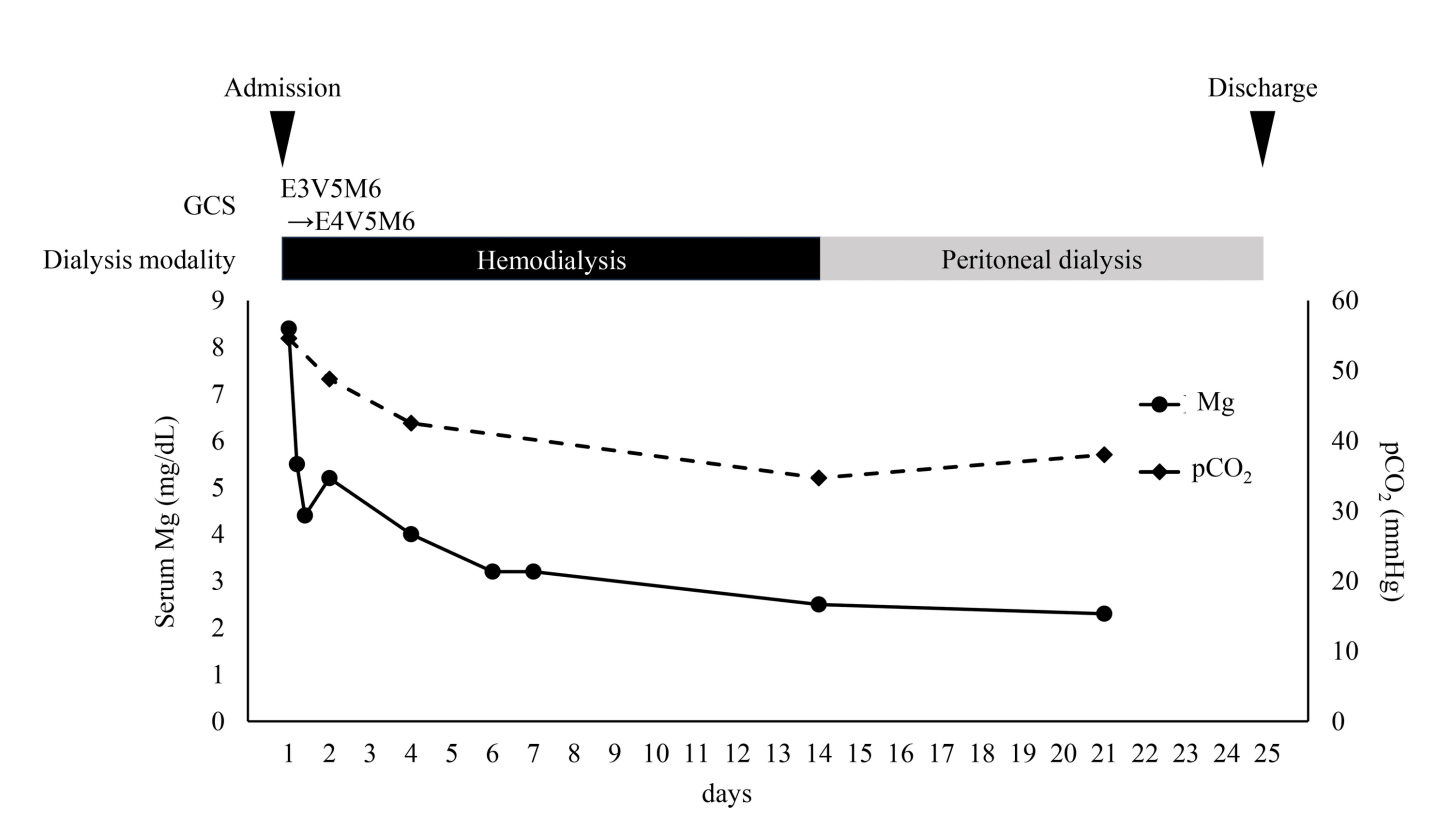
The clinical course of the patient GCS, Glasgow Coma Scale; Mg, magnesium; pCO_2_, partial pressure of carbon dioxide

After being transferred to our department, uremic encephalopathy was suspected due to decreased urine output and elevated serum creatinine levels. Therefore, the treatment was switched from PD to hemodialysis (Figure 2).

Information from a previous hospital revealed that magnesium oxide (1980 mg/day) had been prescribed for constipation during hospitalization. Additional laboratory tests revealed a serum magnesium level of 8.4 mg/dL, which led to a diagnosis of hypermagnesemia-induced encephalopathy and respiratory depression. During hemodialysis, the patient regained consciousness, and his dyspnea resolved. After three hours of hemodialysis, the serum magnesium level decreased to 4.4 mg/dL. The following day, venous blood gas analysis showed improvement, with the pCO₂ reduced to 48 mmHg. Magnesium oxide was discontinued, and serum magnesium levels normalized with continued hemodialysis. Hemodialysis was discontinued on the 14th day of hospitalization, and APD was resumed on the 15th day of hospitalization. The patient did not experience a recurrence of hypermagnesemia, disturbances of consciousness, or respiratory issues. He was discharged on the 25th day of hospitalization and continued rehabilitation and PD on an outpatient basis.

## Discussion

This report discusses the case of a patient undergoing PD who developed severe hypermagnesemia due to the use of a magnesium-containing laxative that subsequently improved with hemodialysis. Serum magnesium levels in patients undergoing PD are influenced by residual renal clearance, PD clearance, and nutritional status, leading to considerable interpatient variability [5]. Although hypomagnesemia has been reported to be associated with overall mortality in patients undergoing PD, hypermagnesemia does not show this association [5,6]. Thus, while hypomagnesemia in this patient population has received considerable attention, hypermagnesemia has not been sufficiently explored. However, severe hypermagnesemia can be fatal and must be carefully monitored. In patients with PD, 18% have been reported to present with hypomagnesemia (serum magnesium level, <1.8 mg/dL) and 19% with hypermagnesemia (serum magnesium level, ≥2.4 mg/dL), with most patients falling within the normal range [5]. However, other studies have reported that 27-70% of patients undergoing PD exhibit hypermagnesemia [3,7]. As hypermagnesemia frequently remains asymptomatic unless it becomes severe, a substantial number of patients undergoing PD may develop subclinical hypermagnesemia. Consequently, any additional factors that increase the serum magnesium levels in these patients could precipitate severe hypermagnesemia, as observed in this case.

Both residual renal function and PD contribute to the clearance of serum magnesium, with residual renal clearance being particularly important [8]. Indeed, the correlations between serum magnesium levels and residual renal clearance have been found to be stronger than those with peritoneal Kt/V [7]. In this case, the patient’s diminished residual renal function likely resulted in reduced magnesium clearance. Although magnesium-based laxatives are inexpensive and widely used, they may induce severe hypermagnesemia in patients with impaired renal function owing to decreased drug clearance. In patients with PD with significantly compromised residual renal function, peritoneal magnesium clearance may not adequately compensate for this reduction, necessitating the cautious use of magnesium-containing laxatives. Notably, two similar cases have been reported in which patients undergoing PD developed severe hypermagnesemia requiring emergency hemodialysis, and both cases were attributed to magnesium-containing laxatives [9,10]. Therefore, when using magnesium formulations as laxatives in patients undergoing PD, it is crucial not to overestimate the clearance achieved by PD but rather to consider alternative agents or to monitor serum magnesium levels meticulously.

## Conclusions

In summary, this case highlights the potentially fatal outcomes associated with the use of magnesium-based laxatives in patients with PD, especially in those with significantly diminished residual renal function. When using magnesium formulations as laxatives in patients undergoing PD, it is crucial to consider alternative agents or to monitor serum magnesium levels meticulously.
